# Pregnancy and the global disease burden

**DOI:** 10.1186/s12978-017-0420-4

**Published:** 2017-12-14

**Authors:** Barbara J. Sina

**Affiliations:** Fogarty International Center, National Institutes of Health, Bethesda, MD USA

**Keywords:** Pregnancy, Ethics, Research, Infections, Non-communicable

## Abstract

Pregnant women experience unique physiological changes pertinent to the effective prevention and treatment of common diseases that affect their health and the health of their developing fetuses. In this paper, the impact of major communicable (HIV/AIDS, tuberculosis, malaria, helminth infections, emerging epidemic viral infections) as well as non-communicable conditions (mental illness, substance abuse, gestational diabetes, eclampsia) on pregnancy is discussed. The current state of research involving pregnant women in these areas is also described, highlighting important knowledge gaps for the management of key illnesses that impact pregnancy globally.

There is a critical need for research with pregnant women, a population long neglected by biomedical investigation. Pregnant women can face significant diseases during the course of their pregnancy, and importantly, pregnancy brings unique physiological changes pertinent to the effective prevention and treatment of these conditions. Appropriate care for pregnant women living with serious illness requires sufficient research to ensure that the health needs of women and their future offspring are met.

## Lack of evidence base for medication use during pregnancy

Despite the need for medication use during pregnancy, most pharmaceuticals lack specific approval for use during pregnancy in the United States or elsewhere. Many pregnant women are treated for serious non-pregnancy related conditions with prescription medications for which there is little evidence for safety and effective dosing in pregnancy. Of more than 450 drugs approved by the US Food and Drug Administration (FDA) between 1980 and 2000, 91% were classified as having “undetermined” teratogenic risk [1]. The average time for a medication to be categorized for risk in pregnancy is 27 years after market approval in the United States [2].

This is not to say that women do not use medication during pregnancy. In 2008, more than 80% of pregnant women in the United States reported use of at least one prescription or non-prescription medication [3]. Commonly prescribed medications for pregnant women include asthma medications, anti-hypertensives, anti-nausea medications, and antibiotics to treat urinary tract infections. In the absence of empirical evidence for most of these drugs, medical providers in the United States routinely depend on case reports, pregnancy registries, observational studies, or anecdotal clinical experience to prescribe medications for pregnant women [4]. Importantly, these information resources are often unavailable to physicians making similar decisions in low- and middle-income countries (LMICs).

The enormous and continuous changes in the physiological state of pregnant women impact the safety and efficacy of any medication used during pregnancy. Thus, these physiological changes necessitate research with pregnant women; most data drawn from research with non-pregnant women cannot properly inform treatment decisions for pregnant women. For instance, physiological changes during pregnancy—e.g., 50% increase in kidney glomerular filtration rate, 40–50% increase blood volume, alteration in serum binding proteins—change drug metabolism such that research findings for non-pregnant women regarding dose, schedule, and toxicity are not generalizable to pregnant women [5, 6].

## Pregnancy research needs: Immunization, infectious disease, and non-communicable disease

To illustrate the need for research with pregnant women, examples of research gaps in important disease states relevant to pregnant women are described.

### Immunization

Dramatic hormonal changes during pregnancy—along with decreased lung capacity, urinary stasis, and change in blood flow—modulate immune responses and may increase infection severity, especially in the third trimester [7], indicating the need for pregnancy-specific data for vaccinations. Immunization during pregnancy can protect the pregnant woman against vaccine-preventable infections as well as protect the fetus and neonate via antibodies transferred from the woman during pregnancy. Vaccination of pregnant women may also provide more overall benefit in some LMIC settings where infectious disease screening and treatment is impractical given limited resources and access to healthcare. For example, group B streptococcus vaccination in South Africa was found to prevent previously high rates of neonatal sepsis and meningitis often untreatable in many communities [8].

Despite the recognized benefits and extensive evidence for vaccine safety [9], theoretical safety issues were the main concern resulting in vaccine refusal by pregnant women globally as well as knowledge regarding vaccination during pregnancy among LMIC healthcare workers [10].

### HIV/AIDS

Pregnancy increases the risk of HIV transmission to women by two-fold [11]. A new HIV infection during pregnancy increases the odds of maternal-to-child transmission by 15-fold [12]. A substantial research evidence base exists for use of antiretroviral drugs for the prevention of mother-to-child HIV transmission, including many clinical trials conducted with HIV-infected pregnant women in LMICs. However, new antiretroviral dosing studies for pregnant women lag behind the clinical use of these new drugs in which dosing was studied in non-pregnant individuals and toxicity is not known. HIV preventatives are also understudied in pregnant women. Most microbicide trials excluded pregnant women [13, 14] with the exception of one study [15, 16]. However, tenofovir gel trials served as a basis for a step-by-step framework developed for research on HIV prevention agents during pregnancy and lactation [17].

### Tuberculosis

Tuberculosis (TB) is a leading cause of maternal mortality in LMIC settings with a high prevalence of HIV [18]. The risk of TB infection increases with HIV coinfection [18]. While the safety and efficacy of first line TB drugs has been established for pregnant women, management of HIV co-infection is complex because rifampicin and isoniazid reduce plasma concentrations of commonly used antiretroviral drugs [18]. Despite the high levels of multidrug resistant TB strains worldwide, there is limited data on the safety of second-line drugs during pregnancy and pregnant women have been excluded from trials of new classes of anti-tuberculosis drugs [19].

### Malaria

Malaria is associated with maternal and neonatal morbidity and mortality. High levels of parasite resistance to the antimalarial drugs currently used for pregnant women and lack new drugs approved for first trimester use are barriers to plans for mass drug administration to eliminate malaria worldwide [20]. The treatment of cases of malaria in pregnancy has lagged years behind the treatment given to non-pregnant patients. Despite observational studies of large numbers of first trimester pregnant women after use of artemisinin-based combination therapy (ACT) demonstrating safety, the World Health Organization has not yet approved ACT to replace quinine [21]. In addition, ACT pharmacokinetics and treatment failure rates suggest that recommended doses, based on insufficient research, are inadequate to treat malaria in second and third trimester pregnant women [22].

### Helminth infections

Widespread hookworm infections in LMICs put pregnant women at greater risk of severe anemia and higher mortality as well as contributing to reduced infant birth weight and increased mortality [23]. Women of reproductive age are currently excluded from public health mass drug administration campaigns to control helminth infections in LMICs [23].

### Emerging epidemic viral infections

Recently, viral infections that significantly impact pregnant women have emerged and spread globally. For example, pregnant women with influenza are at increased risk for hospitalization and death and are more likely to deliver preterm and low birthweight infants [24]. During the 2009 H1N1 pandemic, treatment with oseltamivir was recommended; however, due to limited data, higher complication rates occurred in pregnant women because of under-dosing [25].

The case fatality rate for pregnant women in the recent outbreak of Ebola virus in West Africa was estimated to be approximately 90% with near-zero perinatal survival [26]. Some pregnant women received an experimental intervention with convalescent plasma, and ethical approval was obtained for some to be treated with emergency administration of favipiravir, an antiviral drug [26].

More recently, Zika virus has been confirmed as the cause of thousands of cases of microcephaly in babies born from infected mothers as well as other neurological pathology. Vaccinations have begun in an international multi-site Phase II clinical trial testing in healthy, non-pregnant volunteers [27]. Several surmountable barriers were described for developing these vaccines for pregnant women [28].

### Mental illness

Major depressive disorder, one of the most common complications of pregnancy, is associated with poor neonatal outcomes, e.g. preterm delivery and low birthweight, globally [29]. Randomized clinical trials have not been used to test the safety of antidepressant use during pregnancy because pregnant women are typically excluded from these studies; however, a recent, large retrospective cohort study measured various child health outcomes associated with self-reported use of antidepressants among pregnant women during the first trimester [30].

### Substance abuse

Alcohol attributable deaths of women worldwide amount to 4% of their total deaths and 2.3% of their total burden of disease [31]. Substance abuse, such as alcoholism, in pregnant women may result in birth defects, stillbirths, or infants with higher risk for sudden infant death syndrome (SIDS), neurodevelopmental disabilities, and other health issues. To investigate the etiology and pathogenesis of prenatal alcohol exposure in the risk for SIDS and adverse pregnancy outcomes such as stillbirth and fetal alcohol syndrome, the ongoing Safe Passage prospective longitudinal study enrolled approximately 12,000 pregnant women from the U.S. Northern Plains to include American Indian tribal communities and the Cape Colored communities in the Western Cape of South Africa [32].

### Gestational diabetes

Globally, the burden of hyperglycemia during pregnancy is estimated to be 170 cases per 1000 live births [33]. This burden is expected to increase due to the increasing global prevalence of obesity and type 2 diabetes mellitus [33]. More than 90% of cases of hyperglycemia in pregnancy occur in developing countries [33], where the condition is highly underdiagnosed. Pre-existing diabetes as well as gestational diabetes is associated with serious complications for the pregnant woman and the future child related to metabolic disruption and obstructed labor [33].

### Eclampsia

The average prevalence of preeclampsia in LMICs ranges from 1% to 8%, and eclampsia accounts for approximately 333,000 maternal deaths, six million perinatal deaths, eight million preterm births and 20 million low birthweight infants [34]. Women diagnosed with preeclampsia have a four-fold increased risk for maternal death [34]. Research is needed to understand the factors that influence placental blood vessel growth and function for this hypertensive disorder, e.g. to identify blood protein biomarkers that signal onset for early diagnosis, to develop preventive measures, and to understand the long-term effects on women’s health.

## Conclusions

Across settings and disease types, women encounter severe illness during the course of pregnancy or become pregnant during the course of chronic or persistent illness. Given the high disease burden associated with pregnancy, research with pregnant women is needed to safely and effectively address their health needs and those of their future offspring.

## Abbreviations

ACT: Artemisinin-based combination therapy
FDA: Food and Drug Administration
LMICs: Low- and middle-income countries
SIDS: Sudden infant death syndrome
TB: Tuberculosis
